# Later Positivity Reflects Post-perceptual Processes: Evidence From Immediate Detection and Delayed Detection Tasks

**DOI:** 10.3389/fpsyg.2019.00082

**Published:** 2019-01-31

**Authors:** Muwang Ye, Yong Lyu

**Affiliations:** Academy of Psychology and Behavior, Tianjin Normal University, Tianjin, China

**Keywords:** neural correlates of consciousness, immediate detection, delayed detection, visual awareness negativity, later positivity, event-related potential

## Abstract

Electrophysiological recordings are usually used to study neural correlates of consciousness (NCCs). The aim of our present study was to use two detection tasks to dissociate the electrophysiological correlates of visual awareness from the post-perceptual processes. In immediate detection task, participants had to quickly report whether the stimulus was presented after stimulus, whereas in delayed detection task, participants had to put off reporting whether the stimulus was presented after stimulus. The results showed that two previously frequently observed electrophysiological NCCs were observed: visual awareness negativity (VAN) and later positivity (LP). Importantly, the amplitude of VAN over posterior temporal and occipital areas was not influenced by the task manipulation. However, the amplitude of LP over parietal, posterior temporal and occipital areas was influenced by the task manipulation during 650–850 ms. These data suggest that VAN is an early electrophysiological correlates of visual awareness, and LP reflects post-perceptual processes required in reporting perceptual awareness.

## Introduction

What is the biological basis of consciousness? An important way to answer this question is to identify neural correlates of consciousness (NCCs). In NCC studies, researchers commonly use contrastive experimental design comparing event-related potential (ERP) elicited by physically identical stimuli of which participants are aware *vs*. unaware (Koch et al., 2016; Rutiku and Bachmann, 2017). Using this design, many researchers often found two potential electrophysiological NCCs: visual awareness negativity (VAN) and late positivity (LP; Koivisto and Revonsuo, 2010). VAN is a negative amplitude difference. It typically appears around 200 ms after visual stimulus onset at posterior temporal and occipital electrodes (Koivisto and Revonsuo, 2007; Railo et al., 2011; Koivisto and Grassini, 2016). In addition, LP is a positive amplitude difference. It typically appears after about 300 ms at parietal electrodes (Lamy et al., 2009; Salti et al., 2012; Naccache et al., 2016).

However, more and more researchers realized that the result of NCC studies using contrastive experimental designs is not only NCC but also reflects pre-conscious and post-perceptual processing (Bachmann, 2009; Aru et al., 2012; De Graaf et al., 2012). Therefore, NCC would be confounded by pre-conscious and post-perceptual processing in previous studies.

Especially, participants are commonly instructed to report awareness or unawareness of the liminal stimulus in NCC studies. Therefore, the NCC would be easily confounded with post-perceptual processes required in reporting perceptual awareness (Tsuchiya et al., 2015; Koivisto et al., 2016). The aim of our present study was to dissociate the electrophysiological correlates of visual awareness from the post-perceptual processes. So, we used two task conditions that differed in their requirements on reporting. In immediate detection task, participants had to quickly report whether the stimulus was presented after stimulus, whereas in delayed detection task, participants had to put off reporting whether the stimulus was presented after stimulus. If a potential electrophysiological NCC is modulated by the task manipulation, it must occur after awareness has emerged and reflect post-perceptual processes required in reporting perceptual awareness.

## Materials and Methods

### Participants

Nineteen right-handed undergraduates participated in the study. The data sets of two participants had to be excluded, because they reported awareness in less than 25% of the critical trials or more than 75% of the critical trials. In addition, the data from one participant had to be also excluded, because his data did not contain enough trials for computing the ERPs for each condition (at least 35 per stimulus type) after artifact rejection. The remaining sixteen participants (eight males) had a mean age of 21.25 years (*SD* = 2.46). With normal or corrected to normal vision, none of them reported any history of neurological diseases or brain injuries.

### Stimuli and Apparatus

The stimuli were controlled with E-prime software on a monitor with 1024 × 768 pixels resolution and 60 Hz screen refresh rate, and presented on the center of the gray background (22 cd/m^2^). The critical stimulus was a low contrast sinusoidal Gabor patch (4.24 degree in diameter), tilted 45 degree to left. The Michelson contrast of the critical stimulus was 0.05, 0.06, or 0.07, depending participants performance in pre-experimental calibration phase. In addition, the duration of the critical stimulus was 16.67, 33.34, 50.01, or 66.68 ms, depending participants performance in pre-experimental calibration phase.

### Procedure

**FIGURE 1:**
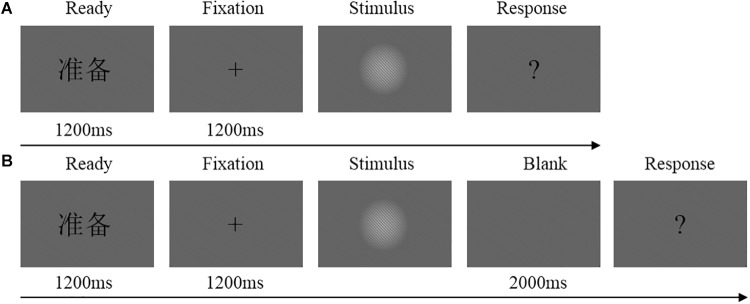
**(A)** Flowchart of experimental trial procedure in the immediate detection task. Each trial started with the presentation of a Chinese word “
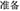
”(i.e., “READY”) at the center of the screen for 1200 ms, flowed by the fixation cross for 1200 ms and the stimulus for an individually calibrated duration (or blank screen in catch trials). After the stimulus (or blank), a question mark was presented until the participants make their decision whether they had seen the stimulus or not. **(B)** Flowchart of experimental trial procedure in the delayed detection task. Each trial started with the presentation of a Chinese word “
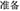
” at the center of the screen for 1200 ms, flowed by the fixation cross for 1200 ms and the stimulus for an individually calibrated duration (or blank screen in catch trials). Then, the screen was blank for 2000 ms. After the blank, a question mark was presented until the participants make their decision whether they had seen the stimulus or not.

Two tasks (immediate detection task and delayed detection task) were performed by each participant in counterbalanced order. Figure 1 shows a flowchart of the trial procedure. The participants were instructed to make their decision whether they had seen the stimulus or not by means of button presses with their left or right index finger. The assignment of seen or unseen to the left or right index finger were counterbalanced across participants.

Both tasks were conducted in two blocks of stimuli, separated by brief resting periods. Half of the participants performed the immediate detection task first (calibration + two experimental blocks), followed by the delayed detection task (calibration + two experimental blocks). Half of the participants performed the tasks in the reversed order. Each stimulus block consisted of eighty critical trials, twenty catch trials, and twenty control trials. The catch trials were no stimuli. In control trials, a higher stimulus contrast (Michelson contrast: 0.08) and a longer stimulus duration (five refresh frames) was used than in the critical trials. During the calibration phase, we used a threshold estimation procedure. For more information regarding the calibration phase (see Koivisto et al., 2016).

### Electroencephalogram (EEG) Recording and Data Analysis

Electroencephalogram was recorded from 64-channel Ag/AgCl electrode cap (Neuroscan, Melbourne, VIC, Australia) with the 10–20 system. The reference electrode was placed on the nose. The ground electrode was placed in front of Fz. EEG was sampled at a digitization rate of 1000 Hz and filtered with a band pass of 0.05–400 Hz. Vertical electrooculogram (VEOG) recording electrodes were positioned above and below the left eye, and horizontal electrooculogram (HEOG) recording electrodes were positioned 1.5 cm from the outer canthus of each eye. The impedance was kept below 5 kΩ.

Electroencephalogram data were analyzed offline with the software of Curry 7. Offline correction of eye movement artifact was performed. To exclude trials contaminated by artifacts, trials with voltages exceeding ±100 μV at any electrode were discarded. The EEG signals were segmented in segmented in series of epochs of 1100 ms. Each epoch started 100 ms before the stimulus onset. Baseline correction was performed over the 100 ms window before the stimulus presentation. The data were filtered with 0.1 Hz high pass and 30 Hz low pass filters. Based on the previous studies (Koivisto and Revonsuo, 2010; Koivisto et al., 2016, 2017) and visual inspection, the mean amplitudes of the ERPs for VAN (250–350 ms) were analyzed (SPSS 22) with repeated-measures analysis of variance (ANOVA) with Awareness (2: aware and unaware), Task (2: immediate detection and delayed detection), Area [2: posterior temporal (P7 and P8), occipital (O1 and O2)], and Hemisphere (2: left vs. right) as factors. In addition, the mean amplitudes of the ERPs for LP (450–650 ms) were statistically analyzed with repeated-measures analysis of variance with Awareness (2: aware and unaware), Task (2: immediate detection and delayed detection), Area [3: parietal (P3 and P4), posterior temporal (P7 and P8), occipital (O1 and O2)], and Hemisphere (2: left vs. right) as factors. The Greenhouse-Geisser correction was applied when the sphericity assumption was violated.

## Results

### Behavioral Results

In critical trials, the participants (*n* = 16) reported awareness in 40.04% (*SD* = 9.35) of the critical trials during the immediate detection task, and in 43.2% (*SD* = 11.49) of the critical trials during the delayed detection task. Moreover, the number of the critical trials with awareness did not differ between the two tasks (*t*_15_ = 0.89, *P* > 0.05). The contrast level of the critical stimulus that participants were tested with was 0.05 Michelson contrast. The duration of the critical stimuli did not differ between the two tasks (*t*_15_ < 0.001, *P* > 0.05).

The participants performed well on the control trials and the catch the trials. They reported awareness in 97.03% (*SD* = 5.18) of the control trials during the immediate detection task, and in 97.03% (*SD* = 6.21) of the control trials during the delayed detection task. In addition, they reported awareness in 4.84% (*SD* = 5.59) of the catch the trials during the immediate detection task, and in 3.91% (*SD* = 6.39) of the catch the trials during the delayed detection task. So, the participants followed the instructions.

### ERPs Results

**FIGURE 2:**
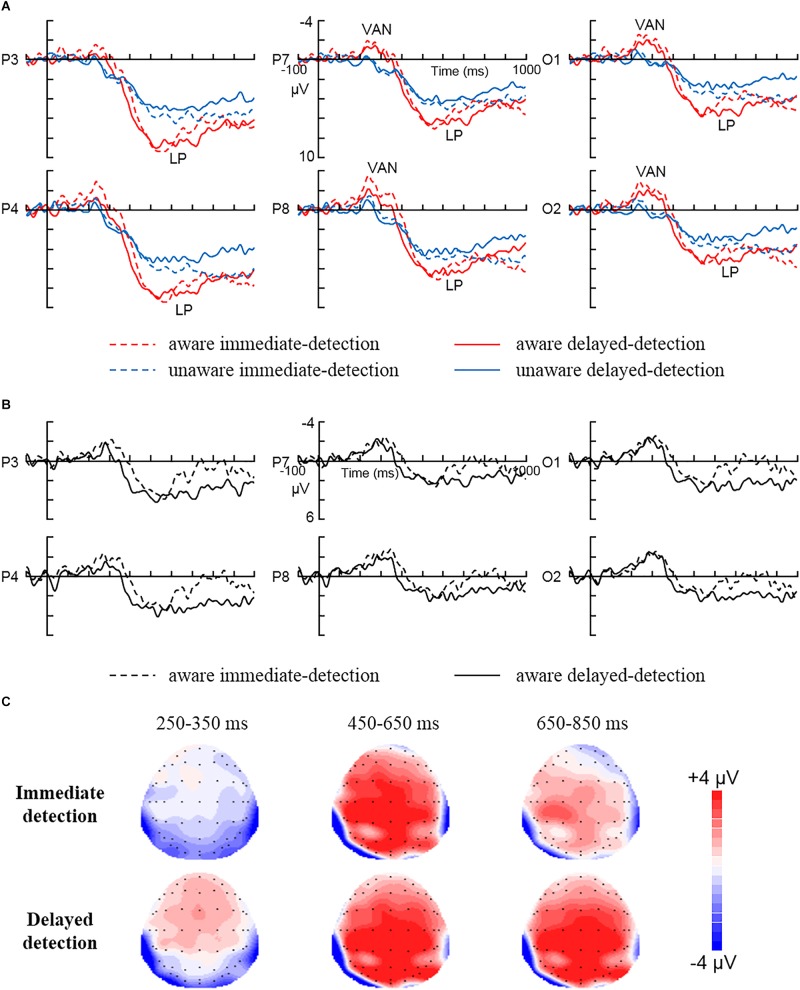
The grand average ERPs for each condition. **(A)** The grand average ERPs to aware immediate-detection, aware delayed-detection, unaware immediate-detection, and unaware delayed-detection in parietal electrodes (P3 and P4), posterior temporal electrodes (P7 and P8), and occipital electrodes (O1 and O2). **(B)** The difference waves (aware minus unaware trials) related to immediate detection and delayed detection over parietal electrodes (P3 and P4), posterior temporal electrodes (P7 and P8), and occipital electrodes (O1 and O2). **(C)** The scalp topography of the mean amplitude difference between aware and unaware during 250–350, 450–650, and 650–850 ms in the immediate detection and delayed detection tasks.

Figure 2 shows the ERP data for each condition. For VAN (250–350 ms), the repeated measures ANOVA showed a significant main effect for Awareness [*F* (1, 15) = 15, *P* < 0.01, $\eta_{p}^{2}$ = 0.5], showing larger negativity in aware trials than in unaware trials (–1.32 ± 0.56 μV vs. 0.53 ± 0.29 μV). However, any other interaction involving Awareness as factor was not statistically significant (*P*s > 0.05).

For LP (450–650 ms), the repeated measures ANOVA showed a significant main effect for Awareness [*F* (1, 15) = 11.25, *P* < 0.01, $\eta_{p}^{2}$ = 0.43], showing larger positivity in aware trials than in unaware trials (6.17 ± 0.9 μV vs. 4.03 ± 0.89 μV). In addition, the Awareness × Area interaction [*F* (2, 30) = 16.41, *P* < 0.01, $\eta_{p}^{2}$ = 0.52] showed that LP was largest over the parietal areas (2.99 μV). However, any other interaction involving Awareness as factor was not statistically significant (*P*s > 0.05). Our results did not show any effects for the task manipulation in the time window of 450–650 ms. However, Figure 2B clearly illustrates that the amplitude of LP decreased to zero during 650–850 ms in the immediate response condition, while the amplitude of LP in the delayed condition did not decrease at all but stayed at the peak level. So, the 650–850 ms time window was also statistically analyzed. The repeated measures ANOVA on mean amplitudes in the time window of 650–850 ms showed a significant main effect for Awareness [*F* (1, 15) = 15.77, *P* < 0.01, $\eta_{p}^{2}$ = 0.51], showing larger positivity in aware trials than in unaware trials (5.34 ± 0.81 μV *vs*. 4 ± 0.81 μV). In addition, the Awareness × Area interaction [*F* (2, 30) = 7.14, *P* < 0.01, $\eta_{p}^{2}$ = 0.32] showed that LP was largest over the parietal areas (1.8 μV). Most importantly, the Awareness × Task × Area interaction was statistically significant [*F* (2, 30) = 3.83, *P* < 0.05, $\eta_{p}^{2}$ = 0.2]. Further, simple simple-effect analysis showed larger positivity in aware trials than in unaware trials during the delayed detection condition over parietal (7.45 ± 1.02 μV vs. 4.55 ± 0.9 μV, *P* < 0.01), posterior temporal (5.29 ± 1.14 μV vs. 3.56 ± 0.98 μV, *P* < 0.05) and occipital (4.51 ± 1.05 μV vs. 2.44 ± 0.88 μV, *P* < 0.05) areas. However, the amplitude changes of the immediate detection condition did not reach statistical significance over parietal (*P* > 0.05), posterior temporal (*P* > 0.05), and occipital (*P* > 0.05) areas. Any other interaction involving Awareness as factor was not statistically significant (*P*s > 0.05).

## Discussion

The results showed that VAN and LP were observed in the immediate detection task and the delayed detection task, respectively. Importantly, our study showed that the amplitude of VAN was not influenced by the task manipulation. VAN was equally strong in the immediate detection task than in the delayed detection task. Thus, our results suggest that VAN correlates with visual awareness. Our finding was consistent with previous studies (Wilenius-emet et al., 2004; Rutiku et al., 2015; Koivisto and Grassini, 2016; Eklund and Wiens, 2018), which suggested that VAN was an early electrophysiological correlate of visual awareness.

In addition, our study showed that the amplitude of LP was influenced by the task manipulation. Our results did not show any effects for the task manipulation during 450–650 ms, but LP was influenced by the task manipulation during 650–850 ms. Both immediate and delayed detection tasks required a similar perceptual decision about the presence or absence of the stimulus. Specifically, working memory need to be updated to map the experience to a response (motor preparation). This may explain why the amplitude of LP during 450–650 ms was similar in both tasks. However, what was different in the tasks was that in the delayed condition overt responding had to be inhibited and the decision kept in memory for the 2 s delay period. LP may correlate with post-perceptual processes such as working memory or perceptual decision (Koivisto et al., 2016; Rutiku and Bachmann, 2017). The delay lengthens the requirement to remember the percept and response; thus, this seems to add or extend a post-perceptual process. This may explain why the amplitude of LP during 650–850 ms was different in both tasks. Thus, our results suggest that LP reflects post-perceptual processing.

Overall, our study adds new data showing that VAN is an early electrophysiological correlates of visual awareness, and LP reflects post-perceptual processes required in reporting perceptual awareness. The further studies are needed to examine the possibility that VAN, which has been assumed to be an early NCC, might reflect pre-conscious processing.

## Ethics Statement

This experiment was approved by the ethical committee in Academy of Psychology and Behavior, Tianjin Normal University. All participants gave written informed consent in accordance with the 2013 Declaration of Helsinki and were paid for their attendance.

## Author Contributions

Both authors designed and performed the experiment, prepared the materials, wrote the manuscript, and approved the final version of the manuscript for submission. MY analyzed the data.

## Conflict of Interest Statement

The authors declare that the research was conducted in the absence of any commercial or financial relationships that could be construed as a potential conflict of interest.
